# From laboratory to point of entry: development and implementation of a loop‐mediated isothermal amplification (LAMP)‐based genetic identification system to prevent introduction of quarantine insect species

**DOI:** 10.1002/ps.4866

**Published:** 2018-03-12

**Authors:** Simon Blaser, Hanspeter Diem, Andreas von Felten, Morgan Gueuning, Michael Andreou, Neil Boonham, Jennifer Tomlinson, Pie Müller, Jürg Utzinger, Jürg E Frey, Andreas Bühlmann

**Affiliations:** ^1^ Agroscope, Department of Method Development and Analytics Wädenswil Switzerland; ^2^ Swiss Tropical and Public Health Institute Basel Switzerland; ^3^ University of Basel Basel Switzerland; ^4^ Federal Office for Agriculture Swiss Federal Plant Protection Service, Zurich Airport Zurich Switzerland; ^5^ Federal Office for Agriculture Swiss Federal Plant Protection Service Bern Switzerland; ^6^ OptiGene Limited Horsham UK; ^7^ The Food and Environment Research Agency York UK; ^8^ Newcastle University Newcastle upon Tyne UK; ^9^ Agroscope, Department of Plants and Plant Products Wädenswil Switzerland

**Keywords:** loop‐mediated isothermal amplification, plant health inspections, point‐of‐entry diagnostics, quarantine organisms, evaluation

## Abstract

**BACKGROUND:**

Rapid genetic on‐site identification methods at points of entry, such as seaports and airports, have the potential to become important tools to prevent the introduction and spread of economically harmful pest species that are unintentionally transported by the global trade of plant commodities. This paper reports the development and evaluation of a loop‐mediated isothermal amplification (LAMP)‐based identification system to prevent introduction of the three most frequently encountered regulated quarantine insect species groups at Swiss borders, Bemisia tabaci, Thrips palmi and several regulated fruit flies of the genera Bactrocera and Zeugodacus.

**RESULTS:**

The LAMP primers were designed to target a fragment of the mitochondrial cytochrome c oxidase subunit I gene and were generated based on publicly available DNA sequences. Laboratory evaluations analysing 282 insect specimens suspected to be quarantine organisms revealed an overall test efficiency of 99%. Additional on‐site evaluation at a point of entry using 37 specimens performed by plant health inspectors with minimal laboratory training resulted in an overall test efficiency of 95%. During both evaluation rounds, there were no false‐positives and the observed false‐negatives were attributable to human‐induced manipulation errors. To overcome the possibility of accidental introduction of pests as a result of rare false‐negative results, samples yielding negative results in the LAMP method were also subjected to DNA barcoding.

**CONCLUSION:**

Our LAMP assays reliably differentiated between the tested regulated and non‐regulated insect species within <1 h. Hence, LAMP assays represent suitable tools for rapid on‐site identification of harmful pests, which might facilitate an accelerated import control process for plant commodities. © 2018 The Authors. *Pest Management Science* published by John Wiley & Sons Ltd on behalf of Society of Chemical Industry.

## 1 INTRODUCTION

The unintended spread of invasive insect species by global trade leads to considerable economic losses in agriculture.^1^, ^2^, ^3^ Numerous insect species have been introduced into Europe, including harmful plant pests such as the western corn rootworm (*Diabrotica virgifera*) and the Colorado potato beetle (*Leptinotarsa decemlineata*).^1^ As global trade is increasing, it is conceivable that the number of successful invasions of plant pests, as well as the scale of their impact, will also increase.^4^, ^5^ Invasive insects can be carried along with imported commodities such as agricultural goods, ornamental plants, nursery stocks, cut flowers, wooden products and packaging materials.^2^, ^6^, ^7^ In addition, pests can unintentionally be vectored as stowaways in transport vehicles (e.g. ships, trains, and lorries), which assist the dispersal along trade networks, including anthropogenic corridors such as canals and railways.^2^, ^8^, ^9^ Besides trade, international tourism, as well as changes in climate and land use also govern the movement of invasive species.^10^

International agreements such as the World Trade Organization (WTO) Agreement on the Application of Sanitary and Phytosanitary Measures (SPS) and the International Plant Protection Convention (IPPC) of the Food and Agricultural Organization of the United Nations (FAO) were concluded with the intention to prevent the spread and introduction of invasive species, as well as to promote the adoption of appropriate measures for their control.^1^, ^11^

Within the European Union (EU), economically harmful plant pests, including insects, are regulated as quarantine organisms and are banned from import to the continent based on the European Council Directive 2000/29/EC.^1^, ^12^ This regulation also prevents the spread of such pests within the EU member states.^1^, ^12^ Switzerland as a non‐EU member has ratified the same plant health regulations in the framework of the agreement between the EU and the Swiss Confederation on trade in agricultural products.^13^ Inspections of plant consignments suspected to harbour quarantine organisms at points of entry (POEs), such as airports, seaports or other border controls, represent an important prevention measure against the introduction and movement of agricultural pests.^1^

In Switzerland, import inspections rely on visual examinations of plant products suspected to harbour quarantine organisms. Yet, morphological differentiation between harmful and non‐harmful insects can be difficult. In particular, the early developmental stages (e.g. eggs and larvae) for which morphological keys are missing are challenging.^14^ Suspicious insects are therefore sent to a reference laboratory (Agroscope, Wädenswil, Switzerland) where they are analysed using DNA barcoding, a method that accurately identifies insects without the need for extensive knowledge of morphological taxonomy. For identification by DNA barcoding, part of the mitochondrial gene cytochrome *c* oxidase subunit 1 (*COI*) is amplified and sequenced.^11^, ^15^, ^16^ The resulting signature sequence is then queried against a database containing reference sequences for different species such as the publicly available Barcode of Life Data System (BOLD).^11^, ^17^ Because the method uses DNA instead of morphological characteristics, it can be equally well used for identification of taxa at all life stages.^11^ Unlike traditional morphological identification, DNA barcoding also enables the identification of cryptic insect pest lineages.^18^, ^19^ However, although barcodes exist for well over 2 million different arthropod species, the method is limited by the fact that it can only identify specimens for which pre‐existing reference barcode sequences are readily available.^11^, ^17^

The shipment of samples to the Agroscope reference laboratory and the subsequent DNA barcoding analysis generally require 2–3 working days. This represents a major drawback of genetic diagnosis, as, in the meantime, the tested import consignments are blocked at the POE. Considering the fact that plant imports often are perishable commodities (e.g. fruits), the import delay due to the time between sampling and diagnosis can result in substantial economic losses for the importer. A promising approach to circumvent this delay is the use of rapid molecular on‐site tests for species identification directly at the POE. The requirements for such an on‐site identification system are, however, considerable. In addition to the feasibility of a test being performed rapidly by plant health inspectors with minimal laboratory training, high diagnostic specificity (true‐negative rate) and sensitivity (true‐positive rate) are pivotal to prevent the import of quarantine insect species and to meet obligations to the trade operators.

Loop‐mediated isothermal amplification (LAMP) is a suitable technology for on‐site analyses of organisms for which taxon‐differentiating DNA or RNA sequences are known.^20^ LAMP is highly specific as this method uses six primer pairs recognising eight distinct DNA regions.^21^, ^22^ Because of its isothermal nature and the robustness against inhibitors, LAMP tests can be performed in a simple and rapid manner in a laboratory‐free environment.^22^, ^23^, ^24^

This paper reports on the development and evaluation of a LAMP‐based identification system for quarantine insects and its successful implementation at the POE at Zurich Airport, Switzerland. The assay allows the molecular on‐site identification of *Thrips palmi*, *Bemisia tabaci*, and several regulated fruit fly species from the genera *Bactrocera* and *Zeugodacus*. The fruit fly assay includes a group of members of the *Bactrocera dorsalis* species complex (*Bactrocera cacuminata*, *Bactrocera carambolae*, *Bactrocera dorsalis*, *Bactrocera papayae*, and *Bactrocera philippinensis*, hereafter the ‘*B. dorsalis* group’), as well as *Bactrocera latifrons* and *Zeugodacus cucurbitae*. These pest species were chosen as targets, because they account for >70% of the intercepted quarantine insect species over the past several years at the POE at Zurich Airport. The reported method has been designed for application by plant health inspectors with minimal laboratory training and can be performed within 1 h. As a result of its simplicity and the speed with which LAMP assays enable precise molecular diagnostics, this method represents a timely and promising new tool for National Plant Protection Organizations (NPPOs) and others in need of rapid identification of potential invasive pests on imported plant commodities.

## 2 METHODS

### 2.1 DNA extraction

For *T. palmi*, DNA was extracted from individual adults, for *B. tabaci* it was extracted from larvae and for the fruit flies it was extracted from approximately 1 mm^3^ of larval tissue. For DNA extraction, tissue samples were added to 30 µl of an alkaline lysis solution [600 µm potassium hydroxide (Sigma‐Aldrich Corp., St Louis, MO, USA) and 2 µm Cresol Red (Sigma‐Aldrich Corp.)] and heated to 95 °C for 5 min on a heat block (Thermomixer Comfort; Eppendorf AG, Hamburg, Germany). The DNA extract was used directly for the LAMP reaction without any purification step.

### 2.2 LAMP primer design

LAMP assays for *T. palmi* and fruit flies of the genera *Bactrocera* and *Zeugodacus* were designed using publicly available sequences of an approximately 650‐bp‐long fragment at the 5′ end of the *COI* gene retrieved from the GenBank database.^25^ For *B. tabaci*, as a result of the high level of sequence variation, a sequence fragment located at the 3′ end of *COI* was chosen as the target sequence for the LAMP assay. Primer design was performed using LAMPdesigner version 1.02 (Premier Biosoft International, Palo Alto, CA, USA) and Geneious versions R7–10.^26^

The fruit fly assay is designed as a combined LAMP test comprising one primer set targeting *B. latifrons* and *Z. cucurbitae*, and a second primer set targeting the *B. dorsalis* group (*B. carambolae*, *B. cacuminata, B. dorsalis, B. papayae*, and *B. philippinensis*). In order to simplify the protocol, the assay does not distinguish between the different fruit fly species targeted by the two primer sets. To ensure the specificity of this assay, sequences from the following closely related, non‐target species were included in the primer design: *Anastrepha* spp. (11 species), *Bactrocera* spp. (five), *Ceratitis* spp. (12), *Dacus* spp. (32), and *Rhagoletis* spp. (five).

With the intention to cover the global sequence diversity observed for *B. tabaci* samples, a combined LAMP assay with three slightly different primer sets was designed. Closely related, non‐target species included in the design of this assay were: *Aleurocanthus* spp. (two), *Aleurochiton aceris*, *Aleurodicus dugesii*, *Bemisia* spp. (three), *Neomaskellia andropogonis*, *Tetraleurodes acacia*, and *Trialeurodes* spp. (four).

The *T. palmi* LAMP test consists of only a single primer set and the following non‐target species were included in the design: *Frankliniella* spp. (two), *Cephalothrips monilicornis*, *Scirtothrips* spp. (five), *and Thrips* spp. (two). Primers of all assays described in this study contain degenerated bases; the types and positions of the degeneracies are given in Supporting Information Table S1. They are available as commercial kits (OptiGene Ltd, Horsham, UK).

### 2.3 LAMP assays

LAMP reactions were performed in eight‐well strips or 96‐well plates. The reaction volume was 25 µl, containing 15 µl of Lyse n' Lamp Isothermal Master Mix (OptiGene Ltd), 1.3 µm F3 and B3 primers, 13.3 µm FIP and BIP primers, 6.6 µm loopF and loopB primers and 2.5 µl of sample DNA extract. LAMP reactions were performed using Genie® II (OptiGene Ltd) or a 7500 Real‐Time PCR System (Applied Biosystems, Carlsbad, CA, USA) at 65 °C for 60 min. To determine the LAMP product melting temperature, samples were heated to 98 °C and cooled to 75 °C, while measuring fluorescence in real time.

As a negative amplification control, 2.5 µl of alkaline lysis solution (described above) was added to the reaction instead of DNA extract. Purified polymerase chain reaction (PCR) amplicons generated in the DNA barcoding approach (described below) were diluted to a concentration of 5 x 10^‐3^ ng µl^–1^ in alkaline lysis solution (described above) and a volume of 2.5 µl was used as a positive amplification control. DNA concentrations of the positive amplification controls were measured using a Qubit 3.0 Fluorometer (Thermo Fisher Scientific Inc., Waltham, MA, USA).

### 2.4 LAMP implementation and procedure at the POE

**Figure 1:**
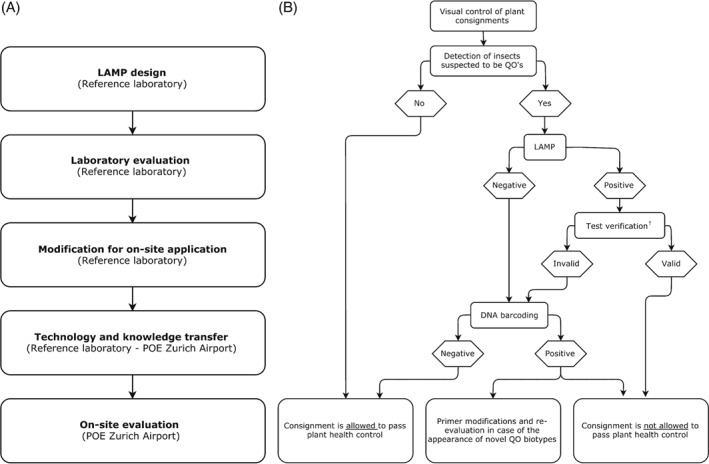
(A) Implementation procedure and (B) workflow of the LAMP‐based identification system at the POE at Zurich Airport. ^†^On‐site test verification was performed by a control application inspecting results of the positive and negative controls, as well as melting temperatures of the LAMP amplification products. POE, point of entry; QO, quarantine organism.

Individual steps in the development, implementation and evaluation of the LAMP assays at the POE at Zurich Airport are illustrated in Fig. 1A. After LAMP primer design, assays were evaluated for diagnostic accuracy under laboratory conditions by testing quarantine insect species intercepted between 2012 and 2015 at the POE at Zurich Airport and results were cross‐validated by DNA barcoding. Thereafter, the LAMP protocol was further adapted to enable plant health inspectors with minimal laboratory training to successfully perform the method under on‐site conditions. The resulting simplified protocol consists of only one single pipetting step, which has been achieved by the fabrication of pre‐mixed LAMP kits, including all chemicals for the DNA amplification reactions. Furthermore, chemicals were stained with a dye (i.e. Cresol Red) to facilitate the handling of the small amount of liquid with the pipette (i.e. by enabling visual checking). LAMP kits were supplied by the Agroscope reference laboratory and stored at ‐20 °C.

After the technology transfer including the installation of a LAMP work station at Zurich Airport, plant health inspectors received basic laboratory training. Subsequent to the first LAMP round supervised by one of the investigators, plant health inspectors performed the LAMP tests independently. In order to evaluate the performance of the implemented identification system, LAMP results from the POE at Zurich Airport were cross‐validated by DNA barcoding.

The workflow of the established identification system consists of visual inspections of incoming plant commodities followed by molecular identification using the LAMP assays in the case of the detection of insects suspected to be quarantine organisms (Fig. 1B). Each LAMP read‐out is then checked for validity using a custom‐written Microsoft® Excel® 2013 application available upon request from the corresponding author. The application checks the presence of amplification, the results of the controls and the expected melting temperature. The following lower and upper melting temperature threshold values were set: fruit fly assay, 80 and 85 °C; *B. tabaci* assay, 80 and 85.8 °C; and *T. palmi* assay, 78 and 84 °C. In the case of a valid positive result, the plant health inspector in charge can immediately destroy or reject the infested cargo.

In the case of a negative or invalid positive result, the DNA extract is sent to the Agroscope reference laboratory and is identified to species level through DNA barcoding. This control step ensures maximum test sensitivity, also preventing the introduction of unknown biotypes not included in the initial primer design. Such unknown biotypes can pose a risk for false‐negative LAMP results, because the DNA amplification‐based identification approach recognises only predefined targets. The addition of a sequencing step in the procedure also allows updating of the current LAMP assays by including new biotypes in the current LAMP primer set.

### 2.5 Analyses of diagnostic accuracy

In order to assess diagnostic accuracy, the following formulas were used to calculate sensitivity (true‐positive rate), specificity (true‐negative rate), positive predictive value (percentage of results that are true‐positive), negative predictive value (percentage of results that are true‐negative), and test efficiency (percentage of correct test results):

$$\text{Sensitivity}\;\left(\mathrm{SEN}\right)=\frac{N_{\mathrm{TP}}}{N_{\mathrm{TP}}+N_{\mathrm{FN}}}\times 100$$

$$\text{Specificity}\;\left(\mathrm{SPE}\right)=\frac{N_{\mathrm{TN}}}{N_{\mathrm{TN}}+N_{\mathrm{FP}}}\times 100$$

$$\text{Positive predictive value}\;\left(\mathrm{PPV}\right)=\frac{N_{\mathrm{TP}}}{N_{\mathrm{TP}}+N_{\mathrm{FP}}}\;\times 100$$

$$\text{Negative predictive value}\;\left(\mathrm{NPV}\right)=\frac{N_{\mathrm{TN}}}{N_{\mathrm{TN}}+N_{\mathrm{FN}}}\;\times 100$$

$$\text{Test efficiency}\;\left(\mathrm{EFF}\right)=\frac{N_{\mathrm{TP}}+N_{\mathrm{TN}}}{N_{\mathrm{TP}}+N_{\mathrm{TN}}+N_{\mathrm{FP}}+N_{\mathrm{FN}}}\times 100$$

where *N* represents the number of analyses, *N*_TP_ the number of true‐positive results, *N*_TN_ the number of true‐negative results, *N*_FN_ the number of false‐negative results, and *N*_FP_ the number of false‐positive results.

### 2.6 DNA barcoding

All specimens included in the laboratory and on‐site LAMP assay evaluation process were also subjected to DNA barcoding. PCR was carried out on a GeneAmp PCR System 9600 (PerkinElmer Inc., Waltham, MA, USA). The following primer pairs were used to amplify the ‘Barcode of Life’ fragment (i.e. the 5′ end of the *COI* gene) of *T. palmi* and the fruit fly specimens: Ron (GGAGCTCCTGACATAGCATTCCC) and C1‐N‐2353 (GCTCGTGTATCAACGTCTATTCC).^27^, ^28^ In order to amplify the barcode fragment of *B. tabaci* located at the 3′ end of the *COI* gene, the primers C1‐J‐2195 (5'‐TTGATTTTTTGGTCATCCAGAAGT‐3') and TL2‐N‐3014 (5'‐TCCAATGCACTAATCTGCCATATTA‐3′) were used.^27^, ^28^ Reactions were run in reaction volumes of 20 µl with 1 × HotStarTaq Master Mix (Qiagen AG, Hilden, Germany), 0.4 µm of each primer and 1 µl of DNA extract diluted 1:10 in molecular grade water. The PCR reaction was performed using the following cycling conditions: 15 min at 95 °C, followed by 45 cycles of 40 s at 95 °C, 15 s at 45 °C, ramping over 60 s to 60 °C and 2 min at 72 °C, and a final elongation step of 7 min at 72 °C. A clean‐up step of the amplification product was performed using the NuceloFast® 96 PCR system (Marcherey‐Nagel GmbH, Düren, Germany).

Linear amplification was carried out on a Labcycler (SensoQuest GmbH, Göttingen, Germany) in 10‐µl reactions containing 1 × BigDye® Terminator v1.1 Ready Reaction Mix (Applied Biosystems), 0.2 µm of either forward or reverse primer (see above) and 1 µl of PCR product diluted 1:10 in molecular grade water. The linear amplification reaction was performed using the following cycling conditions: 15 min at 95 °C, followed by 45 cycles of 15 s at 95 °C, 15 s at 45 °C and 2 min at 72 °C. The DyeEx 96 Kit (Qiagen AG) was used to remove unincorporated dye terminators. The amplicons were then sequenced on a 3130xl Genetic Analyzer (Applied Biosystems) according to the manufacturer's instructions.

Forward and reverse DNA sequences were assembled using Geneious versions R7–10.^26^ The assembled sequences were then blasted for species identification against multiple publicly accessible databases, including GenBank, BOLD and Q‐bank.^17^, ^25^, ^29^ All sequences generated during the on‐site evaluation step were uploaded to GenBank; accession numbers are shown in Supporting Information Table S2.

### 2.7 Sequence analyses

To assess the species‐wide genetic diversity found in the on‐site evaluation samples and to enable estimations of the risk of future false‐negative results, the *COI* sequences of insect specimens analysed during on‐site evaluation were compared to those retrieved from the GenBank database (accessed 15 June 2017). Sequences were aligned with MUSCLE using default parameters implemented in Geneious version 10.0.9.^25^, ^26^, ^30^ To investigate whether the specimens analysed during on‐site evaluation reflect the genetic diversity of larger data sets, genetic diversity indices such as the number of polymorphic sites (*N*_P_), the number of haplotypes (*h*), haplotype diversity (*H*_d_), nucleotide diversity (*π*) and the mean number of pairwise differences (MNPD) were estimated in DnaSP version 5.10.^31^ *In silico* primer specificity analyses were performed using the primer testing function implemented in Geneious version 10.0.9.^26^ Of note, the same software was used to generate pairwise genetic similarity matrices in order to assess the genetic similarity of the on‐site evaluation specimens.^26^

## 3 RESULTS

### 3.1 Primer design and laboratory evaluation of the LAMP assays

The primer sets of the LAMP assays were designed based on the mitochondrial *COI* gene, where *in silico* analyses revealed taxa‐specific regions for the target organisms.

In the first evaluation of the LAMP assays, a total of 282 insect specimens (fruit flies, *N* = 117; *B. tabaci*, *N* = 67; *T. palmi*, *N* = 98) suspected to be quarantine organisms were analysed by LAMP under laboratory conditions (Table 1A). Thereby, the fruit fly assay correctly identified *Z. cucurbitae* specimens from four different countries of origin, *B. latifrons* specimens from two different countries of origin and specimens from the *B. dorsalis* group from nine different countries of origin (Table 2A). Specimens from 13 non‐target, closely related or morphologically similar species gave negative results in the same analysis (Table 2A). During the evaluation of the *B. tabaci* assay, specimens originating from eight different countries were correctly identified and two specimens from a closely related species gave negative results (Table 2B). Of note, the *T. palmi* assay was successfully tested for the identification of specimens originating from eight different countries (Table 2C). The same assay gave negative results when testing eight closely related, non‐target species (Table 2C).

**Table 1:** Results of LAMP assay evaluation performed under (A) laboratory and (B) on‐site conditions at the POE at Zurich Airport

	LAMP assay	*N*	*N*_TP_	*N*_FP_	*N*_TN_	*N*_FN_	SEN (%)	SPE (%)	PPV (%)	NPV (%)	EFF (%)
A	Fruit fly^a^	117	57	0	60	0	100.0	100.0	100.0	100.0	100.0
	*B. tabaci*	67	62	0	2	3	95.4	100.0	100.0	40.0	95.5
	*T. palmi*	98	75	0	22	1	98.7	100.0	100.0	95.7	99.0
	Overall	282	194	0	84	4	98.0	100.0	100.0	95.5	98.6
B	Fruit fly^a^	14	9	0	4	1	90.0	100.0	100.0	80.0	92.9
	*B. tabaci*	13	13	0	0	0	100.0	n/c	100.0	n/c	100.0
	*T. palmi*	10	7	0	2	1	87.5	100.0	100.0	66.7	90.0
	Overall	37	29	0	6	2	93.6	100.0	100.0	75.0	94.6

*N*, number of analyses; *N*_TP_, number of true‐positive results; *N*_FP_, number of false‐positive results; *N*_TN_, number of true‐negative results; *N*_FN_, number of false‐negative results; SEN, sensitivity; SPE, specificity; PPV, positive predictive value; NPV, negative predictive value; EFF, test efficiency; n/c, not calculated.

a The fruit fly LAMP assay includes *B. latifrons*/*Z. cucurbitae*, as well as *the* *B. dorsalis* group (*B. carambolae*, *B. cacuminata*, *B. dorsalis* *, B. papayae,* and *B. philippinensis*).

**Table 2:** Diversity and geographical origin of insect samples used for laboratory evaluation of the LAMP assays for (A) regulated fruit flies of the genera Bactrocera and Zeugodacus, (B) B. tabaci and (C) T. palmi. The B. dorsalis group includes B. cacuminata, B. carambolae, B. dorsalis, B. papayae, and B. philippinensis

	Species	Origin	LAMP		Species	Origin	LAMP
A	*Bactrocera dorsalis* group (5)	Cambodia	+	B	*Bemisia tabaci* (4)	Canary Islands	+
	*Bactrocera dorsalis* group (6)	Cameroon	+		*Bemisia tabaci* (1)	Dominican Republic	+
	*Bactrocera dorsalis* group (8)	India	+		*Bemisia tabaci* (20)	Israel	+
	*Bactrocera dorsalis* group (4)	Malaysia	+		*Bemisia tabaci* (13)	Malaysia	+
	*Bactrocera dorsalis* group (3)	Pakistan	+		*Bemisia tabaci* (14)	Morocco	+
	*Bactrocera dorsalis* group (3)	Sri Lanka	+		*Bemisia tabaci* (1)	Singapore	+
	*Bactrocera dorsalis* group (8)	Thailand	+		*Bemisia tabaci* (9)	Thailand	+
	*Bactrocera dorsalis* group (4)	Uganda	+		*Bemisia tabaci* (3)	Vietnam	+
	*Bactrocera dorsalis* group (1)	Vietnam	+		*Trialeurodes vaporariorum* (2)	Canary Islands	‐
	*Bactrocera latifrons* (3)	Thailand	+				
	*Bactrocera latifrons* (2)	Vietnam	+				
	*Zeugodacus cucurbitae* (3)	Bangladesh	+	C	*Thrips palmi* (9)	Dominican Republic	+
	*Zeugodacus cucurbitae* (1)	Cambodia	+		*Thrips palmi* (16)	India	+
	*Zeugodacus cucurbitae* (3)	The Philippines	+		*Thrips palmi* (1)	Indonesia	+
	*Zeugodacus cucurbitae* (3)	Vietnam	+		*Thrips palmi* (11)	Malaysia	+
	*Anastrepha fraterculus* (3)	Argentina	‐		*Thrips palmi* (19)	Pakistan	+
	*Anastrepha oblica* (3)	Dominican Republic	‐		*Thrips palmi* (10)	Sri Lanka	+
	*Anastrepha* sp. (3)	Dominican Republic	‐		*Thrips palmi* (6)	Thailand	+
	*Anatrichus* sp. (1)	Sri Lanka	‐		*Thrips palmi* (4)	Vietnam	+
	*Atherigona orientalis* (9)	Sri Lanka	‐		*Cephalothrips monilicornis* (1)	Sri Lanka	‐
	*Bactrocera kandiensis* (2)	Sri Lanka	‐		*Frankliniella intonsa* (1)	Vietnam	‐
	*Ceratitis capitata* (5)	Egypt	‐		*Frankliniella occidentalis* (3)	Canary Islands	‐
	*Ceratitis capitata* (2)	Zimbabwe	‐		*Haplothrips* sp. (4)	Thailand	‐
	*Ceratitis cosyra* (7)	Cameroon	‐		*Scirtothrips aurantii* (5)	Swasiland	‐
	*Ceratitis rosa* (1)	Cameroon	‐		*Scirtothrips dorsalis* (1)	Malaysia	‐
	*Dacus ciliatus* (2)	Pakistan	‐		*Thrips parvispinus* (2)	Uganda	‐
	*Drosophila ananassae* (4)	Cameroon	‐		*Thrips tabaci* (5)	Israel	‐
	*Rhagoletis cerasi* (2)	Armenia	‐				
	*Zaprionus indianus* (8)	India	‐				
	*Zaprionus indianus* (8)	Dominican Republic	‐				

The test efficiency of the three individual assays ranged from 95.5% (*B. tabaci* assay) to 100% (fruit fly assay), and an overall test efficiency of 98.6% was calculated (Table 1A). Specificities were found to be 100% for all three tested LAMP assays (Table 1A). The overall test sensitivity was 98.0% and sensitivity was lowest in the *B. tabaci* test (95.4%) (Table 1A). During the first evaluation step, all tests showed a positive predictive value of 100%. A low negative predictive value was assigned to the *B. tabaci* test (40%) because of the low number of true‐negative results (Table 1A). For the fruit fly and *T. palmi* assays, the negative predictive values were found to be 100 and 95.7%, respectively (Table 1A). Altogether, the overall negative predictive value was 95.5% (Table 1A). Mismatches in primer binding sites of false‐negative *B. tabaci* and *T. palmi* biotypes were analysed and primer sets were modified (Table S3). When subsequently re‐tested with the adapted primer sets, samples were correctly identified (data not shown).

### 3.2 On‐site evaluation of the LAMP assays at the POE

A total of 37 insect specimens were analysed by LAMP under on‐site conditions at the POE at Zurich Airport (Table 1B). The overall test efficiency was 94.6% and efficiency ranged from 90.0 to 100% in the individual assays (Table 1B). Specificity was calculated to be 100% for all assays (Table 1B). During on‐site evaluation, sensitivity was lowest in the *T. palmi* assay (87.5%) and an overall sensitivity of 93.6% was calculated. Positive predictive values were found to be 100% for all assays. Negative predictive values for the fruit fly and *T. palmi* assays were 80.0 and 66.7%, respectively (Table 1B). The two false‐negative samples were found to be positive when subsequently re‐tested by the LAMP method in the Agroscope reference laboratory (data not shown). Analysing the pairwise genetic similarity matrix of the DNA barcoding fragment of tested fruit flies, false‐negative *B. latifrons* sample no. 20496 was found to be genetically identical to sample no. 11524, which was correctly identified at the POE (Fig. S1A). The same was true for the false‐negative *T. palmi* sample no. 11535, which was shown to be identical to the correctly identified sample no. 11529 (Fig. S1C).

Test performance of the on‐site evaluation was assessed by analysing the duration until a positive result was available (time to positive) and melting temperatures of amplification products (Table 3). In order to separately investigate test performances of specimens from the *B. dorsalis* group and *B. latifrons*/*Z. cucurbitae*, results of the combined fruit fly assay were stratified (Table 3). Observed average times to positive (mean ± SD) ranged from 33.8 ± 11.6 min (*B. dorsalis* group) to 56.1 ± 5.6 min (*B. latifrons*/*Z. cucurbitae*) (Table 3). The melting temperatures were shown to extend from 80.1 ± 0.4 °C (*T. palmi*) to 82.2 ± 0.4 °C (*B. latifrons/Z. cucurbitae*) and were observed to be very similar for *T. palmi* and the stratified fruit fly samples (Table 3).

**Table 3:** LAMP assay performances under on‐site conditions at the POE at Zurich Airport. In order to investigate LAMP assay performances for individual fruit fly species groups, results of the combined fruit fly assay were stratified for the B. dorsalis group and B. latifrons/Z. cucurbitae

LAMP assay	*N*_TP_	*T*_P_ (min) (mean ± SD)	*T*_M_ (°C) (mean ± SD)
*B. dorsalis* group^a^	6	33.8 *±* 11.6	82.0 *±* 0.3
*B. latifrons/Z. cucurbitae*	4	56.1 *±* 5.6	82.2 *±* 0.4
*B. tabaci*	13	38.4 *±* 10.3	81.9 *±* 0.4
*T. palmi*	8	38.0 *±* 12.5	80.1 *±* 0.4

*N*_TP_, number of true‐positive samples; *T*_P_, time to positive; *T*_M_, melting temperature; SD, standard deviation.

a Includes B. cacuminata, B. carambolae, *B. dorsalis*, *B. papayae*, and B. philippinensis.

### 3.3 Sequence variation at primer binding sites

As a consequence of the lack of genetic information, it is virtually impossible to include the entire taxon‐specific genetic diversity in the evaluation process of genetic tests, at least for non‐model organisms. However, comparative analyses of publicly available sequence information such as from GenBank may estimate how well the on‐site evaluation results reflect the genetic diversity of larger data sets and the risk of producing false‐negative results upon implementation of the methodology. For the following analyses, *B. latifrons* and *Z. cucurbitae* were treated as a single taxonomic unit, enabling estimates of the genetic diversity covered by the primer set of the combined LAMP assay.

The haplotype diversity (± SD) of on‐site evaluation samples was found to be similar for all four species groups and ranged from 0.667 ± 0.204 for *B. latifrons/Z. cucurbitae* to 0.679 ± 0.122 for *T. palmi* (Table 4A). Compared with haplotype diversity values calculated for GenBank sequences (*B. dorsalis* group, *N* = 995; *B. latifrons/Z. cucurbitae*, *N* = 1010; *B. tabaci*, *N* = 2476; and *T. palmi*, *N* = 243), values of on‐site evaluation samples ranged in the same order of magnitude (Tables 4A and B). The highest haplotype diversity (0.832 ± 0.004) was found for *B. tabaci* GenBank sequences (Table 4B). Nucleotide diversity (± SD) and MNPD (± SD) of the airport samples were found to range roughly in the same order of magnitude as nucleotide diversity values from GenBank sequences (Tables 4A and B). An exception was observed for the joint analysis of the two species *B. latifrons* and *Z. cucurbitae* (identified with the LAMP assay targeting both genetically well‐separated species), where tenfold higher values (*π* = 0.106 ± 0.033; MNPD = 10.0 ± 5.8) were detected compared with the GenBank sequences (*π* = 0.019 ± 0.002; MNPD = 1.8 ± 1.0) (Tables 4A and B). The highest values of nucleotide diversity (0.139 ± 0.070) and MNPD (9.8 ± 4.5) for GenBank sequences were found for *B. tabaci* (Table 4B).

**Table 4:** Variability and genetic diversity measures of concatenated LAMP primer binding sites from (A) samples tested during on‐site evaluation and (B) sequences retrieved from the GenBank database

	LAMP assay	*N*	*N*_P_	*h*	*H*_d_ ± SD	*π* ± SD	MNPD ± SD	*L*
**A**	*B. dorsalis* group^a^	6	2	3	0.733 ± 0.155	0.011 ± 0.002	1.1 ± 0.8	103
	*B. latifrons/Z. cucurbitae* b	4	15	2	0.667 ± 0.204	0.106 ± 0.033	10.0 ± 5.8	94
	*B. tabaci*	13	29	6	0.769 ± 0.103	0.086 ± 0.022	8.6 ± 4.3	101
	*T. palmi*	8	8	3	0.679 ± 0.122	0.026 ± 0.023	2.3 ± 1.4	100
**B**	*B. dorsalis* group^a^	995	32	45	0.647 ± 0.016	0.012 ± 0.001	1.2 ± 0.8	103
	*B. latifrons/Z. cucurbitae* b	1010	37	31	0.579 ± 0.012	0.019 ± 0.002	1.8 ± 1.0	94
	*B. tabaci*	2476	70	119	0.832 ± 0.004	0.139 ± 0.070	9.8 ± 4.5	101
	*T. palmi*	243	43	24	0.628 ± 0.030	0.049 ± 0.004	4.9 ± 2.4	100

*N*, number of individuals tested; *N*_P_, number of polymorphic sites; *h*, number of haplotypes; *H*_d_, haplotype diversity; *π*, nucleotide diversity; MNPD*,* mean number of pairwise differences; *L*, sequence length (bp) of analysed sequences SD, standard deviation.

a Includes B. cacuminata, B. carambolae, *B. dorsalis*, *B. papayae*, and B. philippinensis.

b *B. latifrons* and *Z. cucurbitae* were treated as one taxon, because both are identified by the same LAMP assay.

Despite high nucleotide diversity values in the primer binding sites, the designed LAMP primers containing degenerated bases were found to match 100% to all GenBank sequences of *B. tabaci* and the *B. dorsalis* group when tested *in silico*. For *T. palmi*, one mismatch (C/T) was found at position 17 (from the 3′ end) of the B3 primer and two mismatches (C/T) at positions 17 and 20 of the F3 primer (data not shown). Furthermore, primer mismatches at positions 9 (C/T) and 15 (C/G) of the B3 primer were found when analysing GenBank sequences of *B. latifrons/Z. cucurbitae* (data not shown). All described mismatches of *T. palmi* and *B. latifrons*/*Z. cucurbitae* found during *in silico* analyses have been observed in few individual samples during on‐site evaluation at the POE at Zurich Airport without any impact on LAMP performance (data not shown).

## 4 DISCUSSION AND CONCLUSION

From a quarantine perspective, molecular diagnostics methods for the rapid identification of intercepted specimens are crucial to prevent the introduction and spread of morphologically indistinguishable pest species.^10^, ^32^ An ideal identification assay should be fast, reliable, easy to handle, affordable and suitable for on‐site application.^32^ This paper reports the successful development and on‐site implementation of a LAMP‐based system allowing the rapid identification (within 1 h) of three important and frequently intercepted quarantine insect species groups at a POE in Switzerland. The identification system was implemented to be performed by plant health inspectors with minimal laboratory training. The LAMP assays can be performed using simple and affordable equipment and the results are easy to interpret.

DNA amplification‐based technologies such as the LAMP method can only identify specific target DNA sequences.^33^ A comprehensive knowledge of the target sequence diversity is therefore crucial to ensure diagnostic reliability.^34^ Unfortunately, available information is usually very limited for newly emerging quarantine organisms, even more so as import plant commodities originate from all over the world (Table 2). Rare false‐negative LAMP results as a consequence of unknown single nucleotide polymorphisms (SNPs) at the primer binding sites are thus to be expected for all DNA amplification‐based diagnostic tests and any identification system needs to take this into account.

In view of these points, the LAMP identification system for the POE at Zurich Airport was designed as a two‐stage process (Fig. 1B). First, in the case of a positive LAMP result, the plant health inspectors can directly take action to prevent the introduction of the quarantine insect species. Second, in the case of a negative LAMP result, samples are sent to a reference laboratory where they are analysed by DNA barcoding. This procedure ensures maximum diagnostic sensitivity, which is needed to avoid the import of quarantine insect organisms and supports the further development of the LAMP assays in the case of the emergence of unknown insect biotypes.

In a first evaluation step, only four samples (1.4%) from a total of 282 analysed insect specimens gave false‐negative results; all other results were correct. Sequence analyses of the false‐negative samples revealed several new variant SNPs at the primer binding sites. Primer sets were therefore slightly adapted to accommodate these new variants and the modified LAMP assays were successfully revalidated using all available samples.

The evaluation of the LAMP‐based identification system at the POE at Zurich Airport demonstrated that the LAMP assays are reliable for on‐site diagnostics (Table 1B). Indeed, out of 37 analysed insect specimens, only two samples (5.4%) gave false‐negative results and no false‐positive results were identified (Table 1B). DNA sequences of both samples that gave false‐negative results were found to be identical to DNA sequences from true‐positively tested specimens (Figs S1A and C). Furthermore, both samples gave true‐positive results when re‐tested by the LAMP method in the Agroscope reference laboratory (data not shown). This observation suggests that the two identification failures may have been caused by a handling issue during the LAMP assay preparation. However, because negative LAMP results are routinely re‐tested by DNA barcoding in the designed identification system, the import of quarantine insect species would be prevented in both cases.

Future adjustments to further enhance the diagnostic sensitivity could include testing specimens in duplicate and/or including an internal positive control (IPC). The latter measure would allow monitoring of each individual reaction separately and could consist of non‐target control DNA spiked into the initial lysis solution.

During on‐site evaluation, all specimens suspected to be *B. tabaci* were correctly confirmed (Table 1B). This demonstrates how well the plant health inspectors are trained in pre‐identifying regulated insect quarantine organisms. A basic morphological knowledge is indeed crucial to select the appropriate LAMP assay for the identification of suspicious insects. In the case of the *B. tabaci* assay, because of the lack of any negative result during on‐site evaluation, it was not possible to calculate diagnostic specificity and negative predictive value. Monitoring the test performance of this assay will therefore be an ongoing process.

In a comparative analysis, sequences generated during on‐site evaluation were compared to all corresponding sequences currently available from the GenBank database in order to assess whether the observed genetic variability in the primer binding sites reflects the diversity of larger data sets. The nucleotide diversity values of the primer binding sites from the analysed *B. dorsalis* group, *B. tabaci*, and *T. palmi* specimens were found to range in the same order of magnitude as the values calculated for DNA sequences from the GenBank database (Table 4). In contrast, the nucleotide diversity value (0.106) and MNPD (10.0) calculated for the *B. latifrons*/*Z*. *cucurbitae* airport specimens were ten times higher than the values calculated for sequences from the GenBank database. The reason for the observed discrepancy is probably the low sample size, because only two specimens of each of these two genetically well‐differentiated species were analysed (Table 4 and Fig. S1A). Yet, the results of the comparative analysis have to be interpreted with caution because of the relatively small sample size of the on‐site evaluation samples as well as the fact that *B. latifrons* and *Z. cucurbitae* were treated as a single taxonomic unit. Furthermore, the composition of the GenBank entries for a particular species could also be biased because of overrepresentation of certain biotypes as a consequence of focal studies in specific areas.

The results of *in silico* primer specificity analyses revealed that the designed LAMP primers are suitable to detect all known haplotypes from numerous countries of origin of *B. tabaci*, *T. palmi* and several species of regulated fruit flies of the genera *Bactrocera* and *Zeugodacus*. The issue of the high within‐taxon nucleotide diversity has been addressed by the application of degeneracy in primers, as well as the combination of multiple primer sets in the case of *B. tabaci*. Analysing the available sequences from the GenBank database, no primer mismatches were found either for the *B. dorsalis* group or for *B. tabaci*. Only a few mismatches distant from the 3′ end were found for some sequences of *T. palmi* and *B. latifrons*/*Z*. *cucurbitae*. As all observed mismatches were represented in the insect data set that was successfully analysed during the on‐site evaluation at Zurich Airport, they seem to have no influence on the test performance of developed LAMP assays.

Further efforts towards improving the on‐site identification system will focus on (i) expanding the range of diagnostic LAMP assays and (ii) developing on‐site sequencing capabilities to eliminate the need for diagnostic core laboratories. Small next‐generation sequencing‐based systems such as the Oxford Nanopore technology are valuable candidates for on‐site DNA/RNA sequencing.^35^, ^36^ Eventually, a sequencing‐based technology may completely replace diagnostic assays which would eliminate the need for continuous development and evaluation of genetic tests. Furthermore, provided that sequencing is deep enough, information on characteristics such as pesticide resistance genes in arthropods or antibiotic resistance genes in bacteria may be acquired during the same process that identifies the species. Finally, accumulating sequence information of all intercepted specimens, together with the information on the geographical origin, will enable us to reconstruct invasion history in ‘real time’, thus deepening our understanding of how invasive species spread around the globe, enabling the development of new, more sustainable insect pest management strategies.

The successful molecular training of the plant health inspectors during the implementation of the LAMP‐based identification system can be seen as a first step towards the future introduction of a sequencing‐based on‐site identification system. However, until novel sequencing technologies are ready to use for on‐site application, the implemented LAMP assays represent fast and reliable identification tools for quarantine insect species.

## Supporting information


**Table S1.** (Word document, 16.7 KB)
Overview of types and positions of degeneracies used for LAMP primer design. Adenine (A), cytosine (C), guanine (G), thymine (T), M (A or C), R (A or G), W (A or T), Y (C or T).

**Table S2.** (Word document, 12.3 KB)
GenBank accession numbers of partial COI sequences from insect specimens analysed during the on‐site evaluation process.

**Table S3.** (Word document, 13.9 KB)
Primer mismatch analyses of false‐negatively tested laboratory evaluation specimens. Adenine (A), cytosine (C), guanine (G), thymine (T), base pair (bp).

**Figure S1**. (Word document, 23.9 KB)
Pairwise genetic similarity matrices of insect specimens included in the on‐site evaluation with (A) the fruit fly assay, (B) the B. tabaci assay, (C) the T. palmi assay based on a part of the mitochondrial COI gene. Numbers represent percentage of bases which are identical. Fragment lengths: fruit fly assay, 386 bp; B. tabaci assay, 521 bp; T. palmi assay, 364 bp.
