# Supplementary material for: From laboratory to point of entry: development and implementation of a loop‐mediated isothermal amplification (LAMP)‐based genetic identification system to prevent introduction of quarantine insect species
Source: Pest Manag Sci. 2018 Mar 12;74(6):1504–12. doi: 10.1002/ps.4866 (PMC5969315; doi:10.1002/ps.4866)
Supplement: Supplementary file 1 — Table S1. (Word document, 16.7 KB) Overview of types and positions of degeneracies used for LAMP primer design. Adenine (A), cytosine (C), guanine (G), thymine (T), M (A or C), R (A or G), W (A or T), Y (C or T). [file PS-74-1504-s003.docx]

**SUPPORTING INFORMATION Table S1**

Overview of types and positions of degeneracies used for LAMP primer design. Adenine (A), cytosine (C), guanine (G), thymine (T), M (A or C), R (A or G), W (A or T), Y (C or T).
