# Supplementary material for: From laboratory to point of entry: development and implementation of a loop‐mediated isothermal amplification (LAMP)‐based genetic identification system to prevent introduction of quarantine insect species
Source: Pest Manag Sci. 2018 Mar 12;74(6):1504–12. doi: 10.1002/ps.4866 (PMC5969315; doi:10.1002/ps.4866)
Supplement: Supplementary file 2 — Table S2. (Word document, 12.3 KB) GenBank accession numbers of partial COI sequences from insect specimens analysed during the on‐site evaluation process. [file PS-74-1504-s002.docx]

**SUPPORTING INFORMATION Table S2**

GenBank accession numbers of partial COI sequences from insect specimens analysed during the on-site evaluation process.

| **Sample ID** | **GenBank accession number** |
| --- | --- |
| no.11538 | MG727962 |
| no.11514 | MG727963 |
| no.11531 | MG727964 |
| no.11519 | MG727965 |
| no.11512 | MG727966 |
| no.11513 | MG727967 |
| no.20484 | MG727968 |
| no.11549 | MG727969 |
| no.11504 | MG727970 |
| no.20496 | MG727971 |
| no.11524 | MG727972 |
| no.11536 | MG727973 |
| no.11521 | MG727974 |
| no.11520 | MG727975 |
| no.20500 | MG727976 |
| no.20493 | MG727977 |
| no.20492 | MG727978 |
| no.11544 | MG727979 |
| no.11502 | MG727980 |
| no.20499 | MG727981 |
| no.20487 | MG727982 |
| no.20494 | MG727983 |
| no.20491 | MG727984 |
| no.20490 | MG727985 |
| no.11551 | MG727986 |
| no.20488 | MG727987 |
| no.11530 | MG727988 |
| no.11534 | MG727989 |
| no.11545 | MG727990 |
| no.11511 | MG727991 |
| no.11529 | MG727992 |
| no.11535 | MG727993 |
| no.20497 | MG727994 |
| no.11542_1 | MG727995 |
| no.11542_2 | MG727996 |
| no.11543 | MG727997 |
| no.11526 | MG727998 |
