# Supplementary material for: From laboratory to point of entry: development and implementation of a loop‐mediated isothermal amplification (LAMP)‐based genetic identification system to prevent introduction of quarantine insect species
Source: Pest Manag Sci. 2018 Mar 12;74(6):1504–12. doi: 10.1002/ps.4866 (PMC5969315; doi:10.1002/ps.4866)
Supplement: Supplementary file 3 — Table S3. (Word document, 13.9 KB) Primer mismatch analyses of false‐negatively tested laboratory evaluation specimens. Adenine (A), cytosine (C), guanine (G), thymine (T), base pair (bp). [file PS-74-1504-s001.docx]

**SUPPORTING INFORMATION Table S3**

Primer mismatch analyses of false-negatively tested laboratory evaluation specimens. Adenine (A), cytosine (C), guanine (G), thymine (T), base pair (bp).
