# Supplementary material for: From laboratory to point of entry: development and implementation of a loop‐mediated isothermal amplification (LAMP)‐based genetic identification system to prevent introduction of quarantine insect species
Source: Pest Manag Sci. 2018 Mar 12;74(6):1504–12. doi: 10.1002/ps.4866 (PMC5969315; doi:10.1002/ps.4866)
Supplement: Supplementary file 4 — Figure S1. (Word document, 23.9 KB) Pairwise genetic similarity matrices of insect specimens included in the on‐site evaluation with (A) the fruit fly assay, (B) the B. tabaci assay, (C) the T. palmi assay based on a part of the mitochondrial COI gene. Numbers represent percentage of bases which are identical. Fragment lengths: fruit fly assay, 386 bp; B. tabaci assay, 521 bp; T. palmi assay, 364 bp. [file PS-74-1504-s004.docx]

**SUPPORTING INFORMATION Figure S1**

Pairwise genetic similarity matrices of insect specimens included in the on-site evaluation with (A) the fruit fly assay, (B) the *B. tabaci* assay, (C) the *T. palmi* assay based on a part of the mitochondrial COI gene. Numbers represent percentage of bases which are identical. Fragment lengths: fruit fly assay, 386 bp; *B. tabaci* assay, 521 bp; *T. palmi* assay, 364bp.

**A**

**B**

**C**
